# How Stereotactic Radiotherapy Changed the Landscape in Cancer Care

**DOI:** 10.3390/cancers15061734

**Published:** 2023-03-13

**Authors:** Rémy Kinj, Jean Bourhis

**Affiliations:** Service of Radiation Oncology, Department of Oncology, Lausanne University Hospital and University of Lausanne, 1011 Lausanne, Switzerland

The term “stereotactic body radiotherapy” (SBRT) refers to high-precision radiotherapy techniques using numerous beams converging in a small target volume, allowing the delivery of high doses per fraction (>6–7 Gy) in a very few number of fractions [1]. Such high doses per fraction would be toxic and unusable in larger target volumes. In a simplified way, SBRT could be opposed to conventional radiotherapy, using lower doses per fraction delivered in much larger volumes and requiring repeated fractions to locally control the disease (Figure 1). In these cases, fractionation enables the maintenance of acceptable healthy tissue tolerance. To some degree, the remarkable efficacy of SBRT is due to its singular radiobiological properties, adding indirect tumor cell death through vascular damages and antitumor immunity to direct cell death [2]. The growing interest in SBRT in oncology comes from its outstanding results. It represents a non-invasive, highly effective ablative treatment with little to no toxicity; moreover, this can be repeated and sequentially associated with systemic treatments.

SBRT has gradually emerged as an alternative or even as an equivalent treatment to surgical management in several localized cancers. For example, SBRT was progressively introduced as an alternative to conventional radiotherapy or radical prostatectomy in managing localized prostate carcinoma [3,4]. After dozen of clinical trials including hundreds of patients, SBRT is now accepted as a standard treatment for localized prostate cancer, whatever its risk group, from very low to very high-risk groups [5] (Figure 1). However, despite the potential of SBRT, its clinical use in routine practice is limited by the difficulty of generating strong evidence of SBRT compared to surgery. For example, we can mention the early closures of STARS and ROSEL randomized trials that directly compared SBRT to surgery in patients with early non-small cell lung cancer (NSCLC). Both trials were closed due to difficulty in accrual. Indeed, only 58 patients could be randomized between SBRT or lobectomy, and although the results favored SBRT, they were not strong enough to draw definitive conclusions [6,7].

By its ablative approach, SBRT also contributed to distinguishing patients presenting oligometastases with a low tumor burden from patients with multiple metastases with a higher tumor burden. A metastasis-guided approach consisting of ablative SBRT improved overall survival in a well-selected population [8]. This locally curative approach also improved progression-free survival in various types of cancer [9]. However, the definition of oligometastases is debatable [10]. One would like to define a cut-off number of metastases to distinguish oligometastatic disease from more advanced metastatic disease. In contrast, others would prefer to distinguish it depending on the possibility of delivering a curative treatment. Total metastasis volume closely reflects the tumor burden and is likely a good surrogate to assess the possibility of being cured by SBRT. As SBRT allows repeated ablative treatments with minimal toxicity, we can wonder what the real limits of SBRT in the metastatic setting are. Real limitations could be due to factors that would turn SBRT into a more toxic and less effective treatment. Among these, factors such as the oversize of target lesions and rapid tumor growth could represent the real limitations of the curative metastasis-guided SBRT approach. Without such limiting factors, we can guess that SBRT could be repeated as many times as necessary to control the disease, whether or not in association with systemic therapies.

As an example that paved the way for stereotactic radiotherapy in the metastatic setting, its implementation in the central nervous system dramatically changed the outcome of patients with brain metastases. Indeed stereotactic radiotherapy avoided (or at least delayed) palliative whole brain radiotherapy (WBRT) and its disabling side effects, with outstanding local control rates when proper radiation doses are delivered [11]. In parallel, the maximal number of brain metastasis that can be cured by stereotactic radiotherapy has dramatically increased. Furthermore, current stereotactic radiotherapy technics allow an optimal sparing of the normal brain, and the mean brain dose is generally very low, even when treating multiple brain metastases, thus enlarging possibilities to deliver curative stereotactic radiotherapy, which increased from one to more than ten metastases in a decade [12]. However, compared to WBRT, stereotactic radiotherapy has no prophylactic effect, and iterative imaging and repeated stereotactic irradiations are needed to prolong brain tumor control [13] (Figure 2).

Technical evolutions will continue to enhance the accuracy, safety and efficacy of SBRT treatments. When using such high doses per fraction, it is essential to maintain the irradiation margins from tumor to planning target volume as small as possible. Indeed, any increase in the margins will affect the volume of healthy surrounding tissue exposed to high radiation doses, potentially increasing the toxicity. In this frame, lung SBRT may benefit from modern techniques of respiratory gating and tumor tracking that permit the reduction of the total irradiated volume at high doses [14]. New radiotherapy devices may also offer new possibilities for expanding the use of SBRT; as an example, MRI linac-based and proton-based SBRT treatments are under investigation [15,16,17,18].

Finally, the association of SBRT with immunotherapy is under clinical investigation and may also offer new clinical opportunities [19]. For instance, the PULSAR project proposed to modify current fractionations schedules and to deliver a few large dose “pulses” delivered at least a week apart, combined with immunotherapy. This approach could permit personalized SBRT treatments based on noted changes in tumor morphology, location and radiation response [20].

In conclusion, stereotactic radiotherapy is a powerful non-invasive tool for tumor ablation with minimal toxicity. Its use is now rapidly growing in various tumor types and clinical settings. The real limitations of the use of stereotactic radiotherapy are currently under investigation, but it already appears to be an ideal partner in the metastatic setting in combination with systemic treatments.

## Figures and Tables

**Figure 1:**
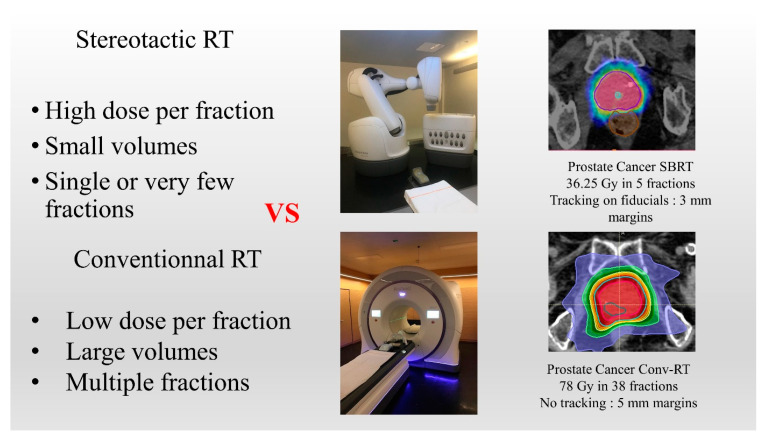
Main differences between stereotactic radiotherapy and conventional radiotherapy.

**Figure 2:**
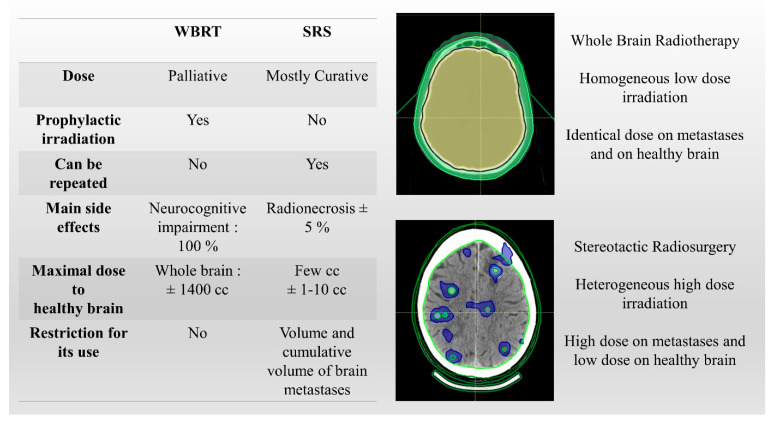
Illustration of the main differences between whole brain radiotherapy (WBRT) and stereotactic radiosurgery (SRS), in upper part WBRT (30 Gy in 10 fractions of 3 Gy, mean brain dose: 30 Gy.), in lower part SRS (3 sequences of stereotactic radiotherapy delivered in a time-lapse of 2 years, 20 Gy in 1 fraction over a cumulative number of 15 metastases from lung cancer, cumulative mean brain dose: 2.6 Gy).
